# Development and Validation of a Simple and Cost-Effective LC-MS/MS Method for the Quantitation of the Gut-Derived Metabolite Trimethylamine N-Oxide in Human Plasma of Healthy and Hyperlipidemic Volunteers

**DOI:** 10.3390/molecules30112398

**Published:** 2025-05-30

**Authors:** Nikolaos A. Parisis, Panoraia Bousdouni, Aikaterini Kandyliari, Maria-Helen Spyridaki, Amalia Despoina Koutsogianni, Christina Telli, Konstantinos K. Tsilidis, Antonios E. Koutelidakis, Andreas G. Tzakos

**Affiliations:** 1 Department of Chemistry, University of Ioannina, 45110 Ioannina, Greece; nparisis@uoi.gr; 2 Department of Food Science and Nutrition, University of the Aegean, 81400 Myrina, Greece; p.bousdouni@gmail.com (P.B.); kandyliari@fns.aegean.gr (A.K.); akoutel@aegean.gr (A.E.K.); 3 Department of Food Science and Human Nutrition, Agricultural University of Athens, 11855 Athens, Greece; 4 General Chemical State Laboratory, Department of Chemical Services of Corfu, Chemical Service of Peloponnese, Western Hellas and Ionian Islands, 49100 Corfu, Greece; marielen.spyridaki@gmail.com; 5 Department of Internal Medicine, Faculty of Medicine, School of Health Sciences, University of Ioannina, 45500 Ioannina, Greece; amaliadespoina.koutsogianni@gmail.com (A.D.K.); tellichr@gmail.com (C.T.); 6 Department of Hygiene and Epidemiology, School of Medicine, University of Ioannina, 45110 Ioannina, Greece; ktsilidi@uoi.gr; 7 Institute of Materials Science and Computing, University Research Center of Ioannina (URCI), 45110 Ioannina, Greece

**Keywords:** TMAO, biomarkers, liquid chromatography, tandem mass spectrometry, method validation, Rhodamine B, human plasma, simple extraction methodology

## Abstract

Trimethylamine N-oxide (TMAO) is a gut microbial metabolite of dietary precursors, including choline and carnitine. Elevated levels of TMAO in human plasma have been associated with several diseases such as cardiovascular, diabetes mellitus, chronic kidney disease, neurological disorders, and cancer. This has led to an increased interest in the accurate determination of TMAO in human blood, for which a reliable, cost-effective and sensitive analytical method should be established. LC-MS/MS has emerged as a powerful tool for the determination of TMAO due to its high sensitivity, specificity, and ability to handle complex matrices. Herein, we describe the development and validation of an LC-MS/MS method for the determination of TMAO in human blood plasma. Our method involves a simple sample preparation protocol, involving a protein precipitation step along with a non-deuterated IS, followed by a Liquid Chromatography-Mass Spectrometry (LC-MS/MS) analysis using a triple quadrupole mass spectrometer. Additionally, the method was adapted and implemented on an UPLC-QTOF/MS. The method was validated using the guidelines set by the European Medicines Agency (EMA) and the US Food and Drug Administration (FDA) for assay performance and robustness in human plasma and successfully applied to plasma derived from healthy and hyperlipidemic volunteers. The developed method was found to be specific, sensitive, and accurate for the determination of TMAO in human plasma, with a lower limit of quantification of 0.25 µM. The intra- and inter-assay precision and trueness were within acceptable limits.

## 1. Introduction

Trimethylamine N-oxide (TMAO) is an amine oxide that is produced from the oxidation of trimethylamine (TMA). This compound is produced by the intestinal microflora in the colon that metabolizes choline, carnitine, betaine, dimethylglycine, ergothioneine, and phosphatidylcholine (precursors). The gut microbiome plays an important role in TMAO metabolism, as certain bacteria can produce high levels of TMA from dietary precursors.

**Figure 1:**
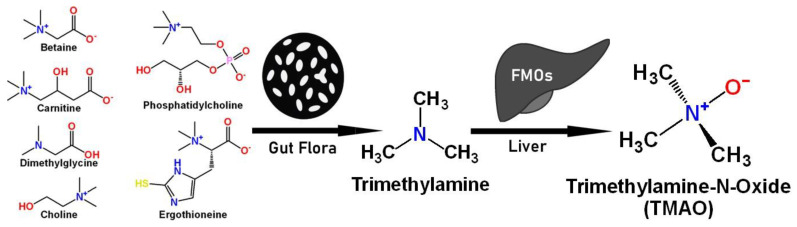
TMA/TMAO precursors and their metabolism to TMAO.

TMAO production in humans begins with the ingestion of nutritional substrates. These compounds are then metabolized by gut bacteria to produce TMA, which is then transported to the liver via the bloodstream. In the liver, TMA is converted into TMAO by the hepatic flavin monooxygenases (FMO1 and FMO3), as shown in Figure 1 [1,2,3,4]. TMAO is then excreted in the urine. Also, TMAO can be absorbed directly from the gastrointestinal tract after the consumption of TMAO-rich foods such as red meat, seafood, or ultra-processed products. In particular, TMAO from fish was able to bypass gut and liver metabolism and enter directly into the blood [5].

Many scientific studies have strongly associated the elevated levels of TMAO to an increased risk of cardiovascular disease (CVD), including atherosclerosis, heart attack, and stroke [6,7,8,9,10,11,12,13,14]. In patients with a moderate or high risk of CVD, a TMAO level of <6.2 µM defines a population at lower risk for Major Adverse Coronary Events (MACE) relative to those with higher TMAO levels. More specifically, a TMAO concentration between 6.2 µM and 9.9 µM indicates a moderately high risk of MACE (2-fold increase at 3 years) relative to those with TMAO levels below 6.2 µM, while a concentration higher than 10 µM indicates a high risk of MACE relative to those with TMAO levels <6.2 µM, given the dose-dependent relationship between TMAO and cardiovascular risk as it had been demonstrated across multiple clinical subgroups [6,10]. A correlation between high plasma concentrations of TMAO and the risk of developing atherosclerosis has been also recorded [9,11,12,13]. Additionally, significantly elevated TMAO levels have been reported in patients with hyperlipidemia [14].

Although TMAO is mainly associated with CVD risk, there are many other studies that associate elevated TMAO levels with other diseases. TMAO levels in blood plasma and serum have been found to be significantly increased in Ischemic Stroke patients [15,16]. TMAO has been associated with chronic kidney disease (CKD) [17] and patients with diabetic CKD showed an increased level of TMAO-producing bacteria, leading to increased TMAO levels in their plasma [18,19]. Another recent study uncovers a bidirectional relationship between TMAO and kidney function [20]. A meta-analysis of 19 prospective studies found that a gradual increase in TMAO was significantly associated with all-cause mortality in patients with CVD, type 2 diabetes (T2D), and CKD, but not in healthy individuals [21]. Higher plasma TMAO levels were associated with stroke severity in diabetic patients [22], liver oxidative stress, inflammation, and fibrosis [23], Abdominal Aortic Aneurysm (AAA) [24], Alcoholic Liver Disease (ALD) [25]. There is also suggestive evidence that TMAO and its precursors have a causal effect on the progression of Parkinson’s disease (PD) [26]. Emerging evidence on the recent coronavirus disease (COVID-19) studies also suggests a link between TMAO and the severity of the disease [27] and it has been revealed that the SARS-CoV-2 nucleocapsid protein amplified the TMAO-induced lipogenesis [28].

Elevated TMAO levels have also been linked to both the natural aging process and the development of neurocognitive disorders; however, more research is needed to clearly understand its role in these processes [29].

To modulate TMAO levels and lower the risks of the diseases linked to them, recent studies are focused on dietary interventions [30], using natural product compounds that have shown great potential in inhibiting TMAO formation [9,31,32].

Given the importance on the determination of TMAO in different diseases, several methods have been developed to detect TMAO in bodily fluids (urine, plasma, serum, cerebrospinal fluid, feces, and saliva) using various methodologies [25,33,34,35,36,37,38,39,40,41,42]. Recent studies also focus on the TMAO direct intake from foodstuff of animal origin, by determining its TMAO content, especially in seafood, where it is widely presented as an endogenous natural ingredient [43,44]. Most of these methodologies [4,34,35,39,40,41,42,45] use a Liquid Chromatography-Mass Spectrometry (LC-MS) standardized protocol [46], where a deuterated trimethylamine-d9 N-oxide solution, diluted in methanol, is utilized as an internal standard (IS), which makes such analyses cost-ineffective. To overcome the high cost, new methodologies are proposed to avoid the use of IS and artificial blood plasma matrix to overcome any recovery issues, but this requires more preparation steps [36].

**Figure 2:**
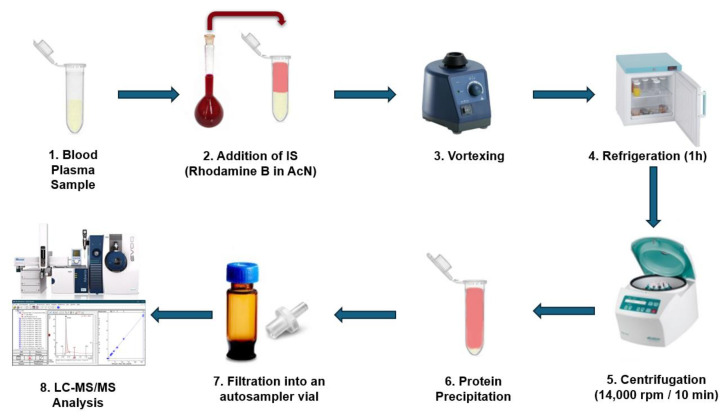
Schematic overview of the experimental workflow.

Herein, we developed a novel UHPLC-ESI-MS/MS method for the determination of TMAO in human plasma. It utilizes Rhodamine B as an IS, a widely available and low-cost compound, that is added to the plasma sample prior to extraction like a surrogate. It has been proven to be an excellent alternative to isotope labeled or other high cost compounds, and provided high recovery results while correcting any variability in the sample preparation procedure. For keeping the analysis simple and cost-effective, we avoided using an artificial plasma matrix. In addition, we used acetonitrile and freezing for better protein precipitation and formic acid for reducing protein binding [47], leading to a rapid preparation protocol that involves just a single protein precipitation step. Including the IS in the precipitating step also reduces the number of steps and errors. This cost- and time-effective methodology has been validated following official guidelines and tried on human blood plasma from healthy and hyperlipidemic volunteers, capitalizing that it can be established as an advantage for applications in clinical trials, for personalized treatments, for primary and secondary prevention, as well as for population risk stratification (Figure 2).

To validate this methodology in human plasma, we followed the official guidelines of the European Medicines Agency (ICH guideline M10 on bioanalytical method validation) [48], US Food and Drug Administration (FDA) [49], Eurachem [50]. The parameters that were evaluated were linearity, sensitivity, accuracy in terms of precision and trueness, selectivity, carry-over, and matrix effect. The method was further applied to a small cohort using plasma samples from hyperlipidemic volunteers. Elevated TMAO levels were detected as anticipated, demonstrating the method’s robustness for cohort-based research applications.

## 2. Results

### 2.1. Optimization of the LC-MS/MS Conditions

The detection of the analyte was performed using an EVOQ Elite ER triple quadrupole (Bruker, Bremen, Germany). For the detection of TMAO and Rhodamine B (IS), positive electrospray ionization (ESI) was performed in Single Reaction Monitoring (SRM) mode. The optimal SRM transition, as well as the fragmentation and ionization parameters, were calculated by direct infusion to the mass spectrometer of a solution containing 500 ng/mL of the analytes at a flow rate of 10 µL/min. The optimal fragmentation and ionization parameters were found to be: spray voltage 4000 V; heated probe gas flow, 50 units; heated probe temperature, 180 °C; cone gas flow, 20 units; cone temperature, 300 °C; nebulizer gas flow, 50 units. The optimal transition of TMAO was found to be m/z 76.40 → 58.60 using a collision energy of 12 eV, and 443.2 → 399.10 at 41 eV for Rhodamine B. Total data acquisition was controlled using MSWS v.8.2.1 software (Bruker, Bremen, Germany).

To further evaluate the ease of translating the methodology to a different MS platform, we also utilized a Xevo G2-XS QToF system (Waters, Milford, MA, USA) for targeted analysis in ToF-MRM mode. The QToF detector settings were set as follows: positive ESI mode; source voltage, 0.5 kV; sampling cone voltage, 5 V; source temperature, 120 °C; disolvation temperature, 600 °C; cone gas flow, 100 L/h; disolvation gas flow, 1200 L/h; declustering potential, 40 V. The optimal transitions of TMAO were found to be m/z 76.08 → 58.07 (quantification) and 59.07 (qualification) using a collision energy of 19 eV, and 443.23 → 399.17 (quantification) and 355.11 (qualification) at 50 eV for Rhodamine B. Total data acquisition was controlled using MassLynx v.4.2 software (Waters, Milford, MA, USA).

### 2.2. Method Validation

Since TMAO is a naturally occurring metabolite that exists in human plasma, it was not feasible to obtain a TMAO-free plasma sample. Thus, this matrix-match method validation was performed using the standard addition methodology, by selecting the plasma sample containing the minimum TMAO amount, which was determined in the process and compensated on the application to real samples. The method was validated according to the guidelines mentioned earlier. Spiked plasma samples were used unless otherwise mentioned.

#### 2.2.1. Linearity and Sensitivity

The linearity of the method was calculated using the IS calibration method and evaluated over a concentration range of 0.25–25.00 µM in three validation runs. For generating the calibration plotting curve, simple linear regression was used for TMAO_area_/IS_area_ against the nominal concentrations of calibration standards (n = 7) (Figure S1). The determined linear equation was Y = 0.3193 × X + 0.0856 with a goodness of fit of R^2^ = 0.9995 (Table 1). It intercepted the *X*-axis at −0.264, thus, the initial concentration of TMAO in the plasma sample was determined to be 0.26 µM.

Experimentally, we set the lower limit of quantitation (LLOQ) at 0.25 µM. Due to the plasma sample already containing an amount of TMAO, we also calculated the theoretical LLOQ based on the standard deviation of the determined concentration of 8 QC samples of 0.25 µM TMAO. It was estimated at 0.22 µM, which was lower than our experimental LLOQ (0.25 µM).

#### 2.2.2. Selectivity and Carry-Over

**Figure 3:**
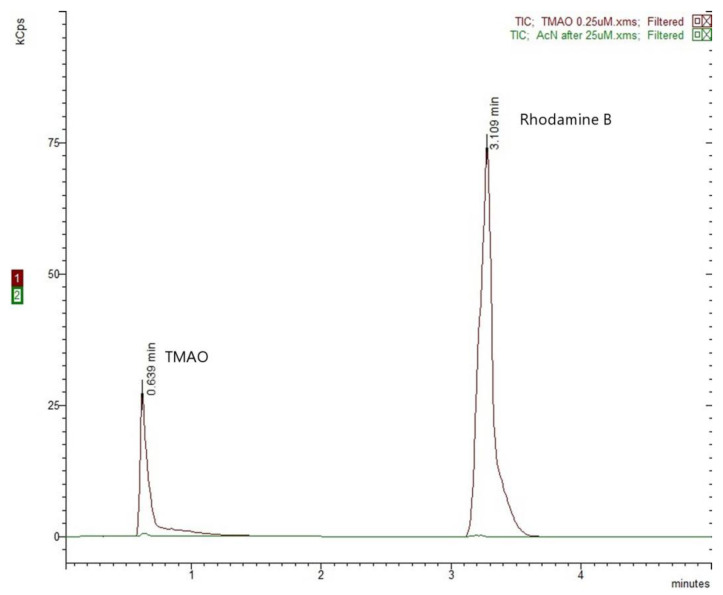
Carry-over assessment: 0.25 µM TMAO chromatogram over pure acetonitrile injection chromatogram analyzed immediately after a high concentration TMAO sample (25.0 µM).

Carry-over assessment was performed by injecting only acetonitrile after the injection of a high concentration sample of 25 µM (upper limit of quantification). The response of the acetonitrile samples at the retention times of the analyte and the IS was negligible relative to the peak areas of the LLOQ sample (0.25 µM TMAO and IS) and therefore no carry-over is observed (Figure 3).

Selectivity was evaluated by analyzing human plasma samples from six different healthy donors to test the interference in the retention times of TMAO and IS of the analysis. TMAO is a naturally occurring metabolite that exists in human blood plasma, so we expected to detect it in the “blank” samples. Five spiked samples with 0.25 µM TMAO from each donor were analyzed, resulting in 30 retention time results.

The chromatograms did not reveal any interfering peaks in the retention times of TMAO and IS (Figure S3). After 30 sequential injections, the average TMAO retention time was 0.637 ± 0.005 min and IS retention time was 3.105 ± 0.021 min. Void time was also calculated and determined as 0.534 min, avoiding any unretained compounds that could interfere with the analyte.

#### 2.2.3. Recovery and Matrix Effect

The percentage of recovery of the method was calculated at four QC levels; 0.25, 1.0, 5.0, and 25.0 µM, by comparing the TMAO/IS responses from plasma samples (n = 5) spiked with TMAO prior to extraction, with responses from plasma samples spiked with TMAO after extraction. The methodology showed a very low relative standard deviation (RSD) and an excellent recovery of the analytes between 98.9% and 105.8% (Table 2).

The percentage matrix effect was determined by comparing the signal of TMAO spiked plasma samples with the mean signal of TMAO samples in 25% acetonitrile in water solution. For this purpose, three replicates at concentrations of 0.5, 5.0, and 25.0 µM were used, and the areas of TMAO were calculated by the MSWS software. The plasma/water TMAO signal percentages were found to be 71.6%, 64.7%, and 66.1%, respectively, indicating about one-third of detector signal suppression from the blood plasma matrix. The matrix effect on the IS was also determined and found to be 74.3%, resulting in about one-fourth of detector signal suppressed by the blood plasma matrix.

#### 2.2.4. Accuracy in Terms of Trueness and Precision

The intra- and inter-day accuracy was determined using spiked plasma samples at four different concentrations of TMAO: LLOQ (0.25 µM), low (1.0 µM), medium (5.0 µM), and high (25.0 µM), with five replicates for each concentration. Inter-day trueness (as % Relative Error—%RE) and precision (as % Relative Standard Deviation—%RSD) were determined on three consecutive intra-day runs (n = 20) (Table 3).

The intra-day trueness and precision were found to be ≤4.8% and ≤10.9%, respectively, while the inter-day accuracy and precision were ≤3.1% and ≤9.6%, respectively. All were within the acceptance criteria (<15%) [48].

### 2.3. Application of the Method to a Pilot Study of Healthy and Hyperlipidemic Volunteers

TMAO levels were analyzed in plasma samples from twelve healthy and seven hyperlipidemic volunteers, comprising both males and females, aged 20 to 71 years. The results showed that TMAO levels in healthy volunteers ranged from 0.666 to 2.882 µM (Table 4), consistent with expected values for healthy individuals. In contrast, hyperlipidemic volunteers exhibited significantly elevated TMAO levels, ranging from 3.553 to 11.847 µM [4,10,14].

**Figure 4:**
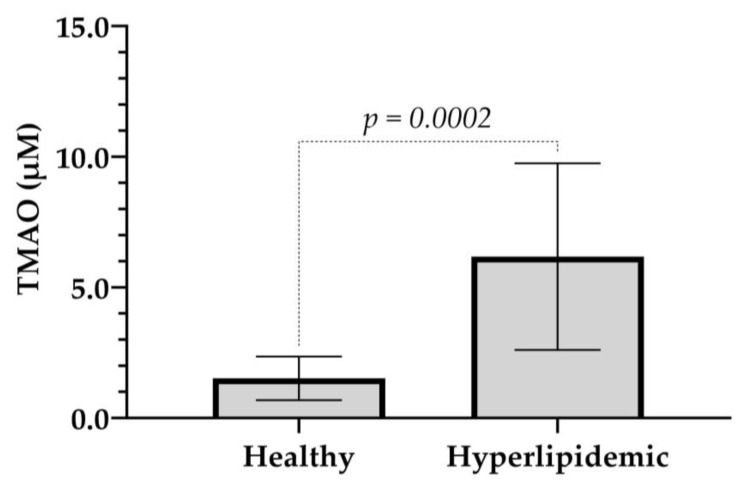
**A** bar graph illustrating the TMAO plasma levels in healthy and hyperlipidemic volunteers. Values represent the mean ± SD. *p* = 0.0002 compared to the healthy group.

These findings further suggest that healthy individuals are more likely to have a favorable lipid profile compared to those with hyperlipidemia. To have a better understanding of this association, we compared the TMAO values of the healthy volunteer group with the ones of the hyperlipidemic. As shown in Figure 4, 6-fold higher TMAO levels were observed in the hyperlipidemic group, with statistically significant difference compared to the healthy volunteers (6.320 ± 3.418 vs. 1.519 ± 0.834, *p* < 0.001).

To validate this, we assessed key biochemical biomarkers in all participants. All plasma samples were also analyzed to determine C-Reactive Protein (CRP), total cholesterol (TC), triglycerides (TG), High-Density Lipoprotein (HDL), and Low-Density Lipoprotein (LDL) cholesterol. These values are presented in Table 4, together with the age, sex (male/female—M/F), and Body Mass Index (BMI) of each participant.

The statistical analysis comparing the hyperlipidemic group to healthy volunteers, based on the data presented in Table 4, demonstrated a statistically significant difference (*p* < 0.05) across all analyzed parameters (Table 5). These findings support the hypothesis that individuals with a favorable lipid profile tend to maintain lower plasma TMAO levels, whereas hyperlipidemic individuals typically exhibit significantly elevated TMAO concentrations [14].

**Figure 5:**
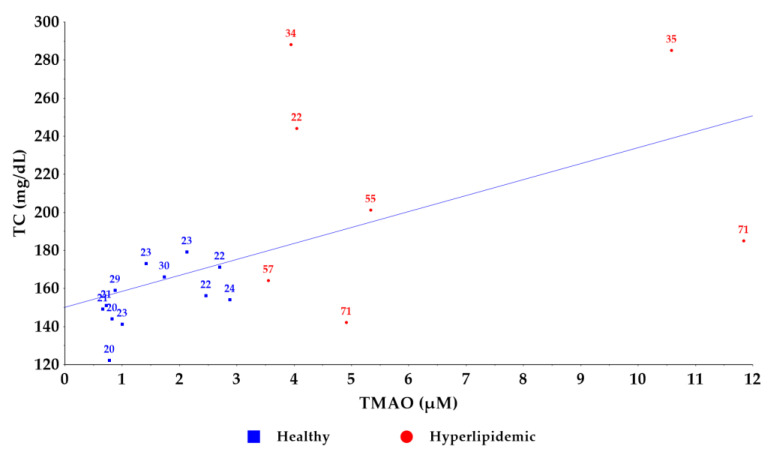
Correlation graph between TMAO and total cholesterol levels in healthy and hyperlipidemic groups. The age of the volunteers is noted on the graph points.

**Figure 6:**
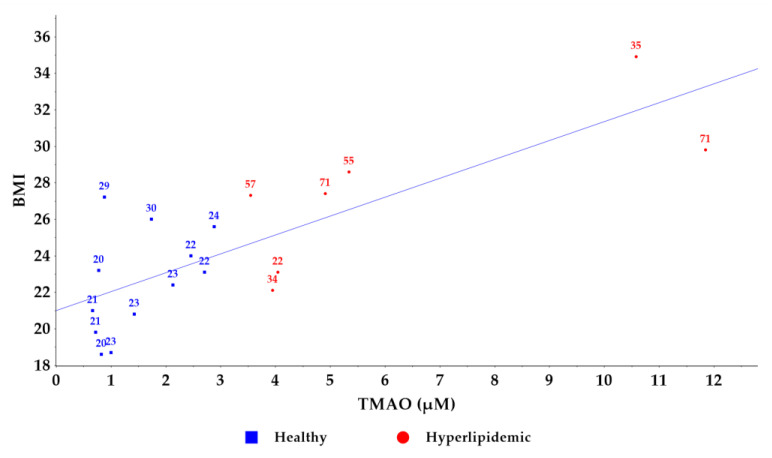
Correlation graph between TMAO and BMI levels in healthy and hyperlipidemic groups. The age of the volunteers is noted on the graph points.

Furthermore, we visualized the relationship between TMAO levels and total cholesterol concentrations (Figure 5). While an increasing trend in cholesterol values was observed with rising TMAO levels, a direct correlation could not be established. Notably, older individuals exhibited higher TMAO levels compared to younger counterparts with similar total cholesterol levels [29]. A similar pattern was observed between TMAO levels and BMI values (Figure 6), supporting previous findings that TMAO concentrations increase with BMI [33]. Despite the small number of samples used to establish the new method, these results confirm the association between circulating TMAO levels in relation to the lipid profile and dyslipidemias with their associated comorbidities [14]; however, further research is needed to elucidate the underlying mechanisms of this relationship.

**Figure 7:**
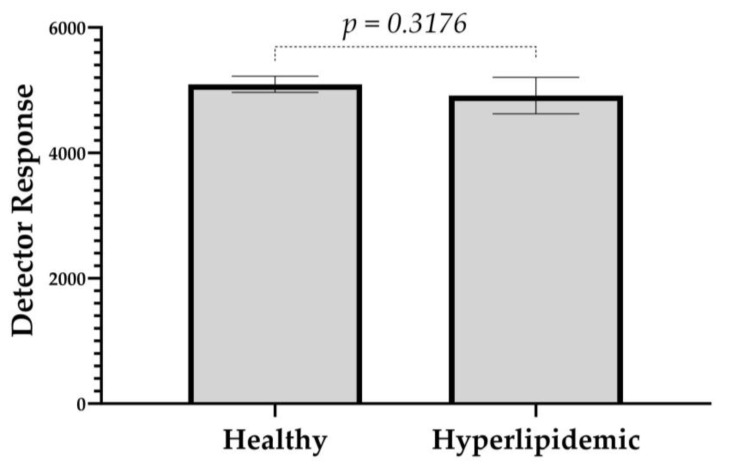
A bar graph illustrating the detector response signals of the IS in plasma samples from healthy and hyperlipidemic donors to evaluate the matrix effect of hyperlipidemic samples. Values represent the mean ± SD. *p* = 0.3176 compared to the healthy group.

The analysis of hyperlipidemic plasma samples was further utilized to evaluate the matrix effect on the IS. A comparison of detector responses of Rhodamine B between healthy and hyperlipidemic plasma revealed no significant variation (Figure 7). This suggests that a higher lipid profile did not interfere with the ionization of the IS, causing signal suppression, possibly because Rhodamine B, as a chloride salt, is already positively ionized. (C_28_H_31_N_2_O_3_^+^ Cl^−^).

### 2.4. Methodology Transfer to UPLC-QToF

The validated methodology was successfully transferred to a different mass spectrometry platform (Waters Xevo G2-XS QToF) to evaluate its reproducibility and ease of implementation. Using the same chromatography conditions on a Waters Acquity i-Class Plus system and optimized mass spectrometry settings, we reanalyzed the calibration standards along with the plasma samples collected from the volunteers (Figure S4). The results demonstrated an excellent calibration curve with a goodness of fit of R^2^ = 0.9997 (Figure S4), while the relative deviation of sample concentrations determined on the QToF compared to the MS/MS results was less than 5%.

## 3. Materials and Methods

### 3.1. Chemicals and Materials

Trimethylamine N-oxide dihydrate was purchased from Merck (Darmstadt, Germany) and Rhodamine B Chloride from Ferak Berlin (Berlin, Germany). Both reagents were of analytical grade (purity > 99.0%). Water, acetonitrile, and formic acid (LC-MS grade) were all purchased from Merck (Darmstadt, Germany). A Kern ABT 120-5DM (Balingen, Germany) 5-digit calibrated analytical balance was used for compound weighing. Before injection, the samples were filtered with 0.2 µm Minisart RC 4 syringe filters from Sartorius (Surrey, UK). Human plasma from healthy donors, used for method development, was generously provided by the Blood Donation Center at the University Hospital of Ioannina.

Plasma samples from healthy volunteers were obtained from the University of the Aegean, Department of Food Science and Nutrition, while plasma samples from hyperlipidemic volunteers were provided by the Lipid Outpatient Clinic of the University Hospital of Ioannina. The study protocol received ethical approval from the local Institutional Ethics Committee, ensuring compliance with established ethical standards. Additionally, informed consent was obtained from all participants.

The plasma samples were also analyzed to determine C-Reactive Protein (CRP), total cholesterol (TC), triglycerides (TG), High-Density Lipoprotein (HDL), and Low-Density Lipoprotein (LDL) cholesterol. University of the Aegean determined all these parameters with a COBAS c111 automated biochemical analyzer (Roche, Basel, Switzerland). At the University Hospital of Ioannina, TC concentrations were determined enzymatically using an Olympus AU600 Clinical Chemistry Analyzer (Olympus Diagnostica, Hamburg, Germany), HDL was measured using a direct assay (Olympus Diagnostica, Hamburg, Germany), while LDL was calculated using the Friedewald formula, and CRP concentrations were assessed with a high sensitivity immunonephelometric assay (Beckman Instruments, Fullerton, CA, USA).

### 3.2. UHPLC-ESI-MS/MS Instrumentation

An Advance™ UHPLC system (Bruker, Bremen, Germany) was used for performing the liquid chromatography separation, utilizing a Kinetex C18 column 100 mm × 2.1 mm, 2.6 µm (Phenomenex, Torrance, CA, USA), set at a temperature of 40 °C. The mobile phase was composed of 0.1% formic acid in LC-MS grade water (A) and 0.1% formic acid in LC-MS grade acetonitrile (B). A constant flow rate of 350 µL/min was used for the following gradient: initial phase (B) concentration 50%, kept constant for 2 min, then increased to 100% within 1 min, kept constant for 1 min, reduced back to 50% in 0.1 min and kept at 50% till the end of the run. The analysis run time was 6 min and the injection volume was set at 1 µL.

For the detection of the analytes, an EVOQ Elite ER triple quadrupole mass spectrometer (Bruker, Bremen, Germany) was used in positive electrospray ionization (ESI) using Single Reaction Monitoring (SRM). The peak area of the analyte versus the peak area of the internal standard was used against the spiked concentration of the analyte to construct the calibration curve and quantify the QC samples. The UHPLC-MS/MS system was controlled using the MS-WorkStation v.8.2.1 SR1 software (Bruker, Bremen, Germany).

To evaluate the potential translation of this methodology to other platforms, we also utilized a UPLC-QToF system, using the Acquity i-Class Plus liquid chromatography assembly tandem to a Xevo G2-XS QToF by Waters Corp. (Milford, MA, USA), and the Waters Masslynx v.4.2 software platform for data acquisition and processing of the results. The chromatography conditions have remained the same.

### 3.3. Preparation of Stock and Working Solutions for the Quantification in Human Plasma

To prepare a 1 M TMAO stock solution, 1.111 g of TMAO dihydrate was dissolved in 10 mL of LC-MS grade water. It was then separated into 10 vials of 1 mL and stored at −20 °C for a maximum period of one month. Each vial was defrosted and used only when needed for preparing fresh working solutions and was discarded afterwards. The standard working solutions were prepared by successive dilutions with water to get final concentrations of 5, 10, 20, 50, 100, 200, and 500 µM. For the QC samples, separate working solutions of 5, 100, and 500 µM were also prepared from a different stock solution. All working solutions were freshly prepared on the day of use. Already published stability studies [46] have been taken into consideration for the selected storage conditions.

Rhodamine B was used as an internal standard (IS); 0.4 mg of Rhodamine B Chloride were diluted in 100 mL LC-MS grade acetonitrile containing 0.1% formic acid, resulting in a 4 mg/L Rhodamine B solution. This solution was kept in a glass container at a constant temperature of −20 °C for a maximum period of one week.

### 3.4. Preparation of Calibration Standards and Quality Control Human Plasma Samples

The calibration standards were prepared in the following concentrations: 0.25, 0.50, 1.0, 2.5, 5.0, 10.0, and 25.0 µM of TMAO. Five microliters (5 µL) of the respective standard working solution were added to 95 µL of drug free human plasma. QC samples with TMAO concentrations of 0.25 µM (lower limit), 5.0 µM (middle), and 25.0 µM (upper limit), were prepared in a similar way.

### 3.5. Human Plasma Sample Preparation

Drug-free human plasma from healthy donors was kindly provided by the Blood Donation Center of the University Hospital of Ioannina. Initially, the plasma samples were allowed to thaw at room temperature. Calibration and quality control standards have been freshly prepared for generating the calibration curves. In 95 µL of the plasma sample, 5 µL of the appropriate TMAO working solution was added. Then, 300 µL of an ice-cold solution of Rhodamine B in acetonitrile containing 0.1% formic acid was added. The samples were mixed vigorously on a vortex mixer and left at 4 °C (refrigerator) for 1 h. Then, the samples were centrifuged at 14,000× *g* for 10 min and 100 µL of the supernatant was transferred to a 1.5 mL Eppendorf tube, where 300 µL of LC-MS grade water containing 0.1% formic acid was added. The final solution was filtered using an RC membrane filter with a pore diameter of 0.2 µm (Sartorius, Surrey, UK), transferred to a 2 mL autosampler glass vial, sealed with a PTFE/silicone cap, and placed in the autosampler, avoiding direct sunlight, at a temperature of 10 °C for immediate analysis (Figure 2).

### 3.6. Method Validation for the Quantification of TMAO in Human Plasma

To validate our methodology in human plasma, we followed the official guidelines of the European Medicines Agency (ICH guideline M10 on bioanalytical method validation) [48], US Food and Drug Administration (FDA) [49], Eurachem [50]. The parameters that were evaluated were linearity, sensitivity, precision (as % Relative Standard Deviation), trueness (as % Relative Error), selectivity, carry-over, and matrix effect. Unless otherwise mentioned, spiked plasma samples were used. The statistical analysis and graph plotting were performed using Prism v10.1.0 for Windows by GraphPad Software and The Unscrambler^®^ X v10.4 by CAMO Software, respectively.

### 3.7. Application to Pilot Study of Healthy and Hyperlipidemic Donors

TMAO analysis was conducted on plasma samples from healthy and hyperlipidemic adults. Blood samples were collected from twelve healthy and seven hyperlipidemic volunteers, both male and female, aged 20 to 71 years. Informed consent was obtained from all participants in accordance with ethical guidelines. Anonymity, confidentiality, and the potential for harm were carefully considered, and all results were appropriately communicated. A comprehensive assessment of patients’ demographic and clinical characteristics was conducted, with a focus on gender, age, body weight, and lipid profile. Healthy volunteers visited the Laboratory of Nutrition and Public Health at the University of the Aegean, while hyperlipidemic volunteers visited the Lipid Outpatient Clinic at the University Hospital of Ioannina. All samples were collected early in the morning after a 12-h fasting period.

Ten milliliters (10 mL) of blood were collected by a cooperating doctor from each volunteer by venipuncture. Blood samples were collected in tubes containing K3 EDTA as an anticoagulant, followed by centrifugation at 3000× *g* at 4 °C for 10 min to isolate plasma. All plasma samples were aliquoted and stored at −40 °C until further analysis.

The analysis of hyperlipidemic plasma samples was further utilized to evaluate the matrix effect on the IS. A comparison of detector responses between low-lipid and hyperlipidemic plasma revealed no significant variation. This suggests that a higher lipid profile did not interfere with the ionization suppression of the IS, possibly because Rhodamine B, as a chloride salt, is already positively ionized. (C_28_H_31_N_2_O_3_^+^ Cl^-^).

## 4. Conclusions

In summary, the developed and validated ultrahigh performance liquid chromatography–electrospray ionization tandem mass spectrometry (UHPLC-ESI-MS/MS) method provides a rapid, simple, sensitive, and accurate alternative for the determination of TMAO in human plasma. The method was successfully validated according to regulatory guidelines by evaluating linearity, sensitivity, precision, trueness, selectivity, carry-over, and matrix effect. All results fulfilled the validation acceptance criteria stated in the European Medicines Agency (EMEA) [48], US Food and Drug Administration (FDA) [49], and Eurachem guidelines [50]. The use of Rhodamine B, an inexpensive synthetic compound, as an internal standard, instead of a deuterated one, proved to be an excellent choice. The results support the suitability, sensitivity, reliability, and reproducibility of this methodology which was successfully applied in human plasma samples obtained from healthy and hyperlipidemic volunteers. These results further elaborate with the premise that healthy people with favorable lipidemic profile are expected to have low TMAO blood plasma values. However, larger studies are needed to ensure these results. This method can serve as a robust and reliable diagnostic tool for clinical and experimental studies addressing the importance of TMAO in population risk stratification and personalized treatments. It is worth noting that the method was developed, validated, and applied to plasma samples from individuals with both favorable and unfavorable lipid profiles. In general, plasma samples with hyperlipidemia could potentially interfere with the quantification of internal standards due to matrix-induced ionization suppression, thereby compromising the accuracy of the methodology. However, during this pilot application, no significant variation was observed between samples from healthy and hyperlipidemic donors. This finding further highlights the robustness of our method for use in cohort studies and supports the selection of Rhodamine B as a valid, cost-effective alternative to deuterated internal standards.

## Figures and Tables

**Table 1:** Method linearity and theoretical lower limit of quantification (LLOQ).

Concentration (µM)	Equation	R^2^	RSD (Mean)	LLOQ
0.25–25.0	Y = 0.3193 × X + 0.0856	0.9995	0.02	0.22

**Table 2:** TMAO recovery.

QC Concentration (µM)	Average (n = 5)	% RSD (n = 5)	% Recovery
0.25	0.25	0.03	98.9%
1.0	1.05	0.03	104.8%
5.0	5.06	0.10	101.3%
25.0	25.28	0.61	101.1%

**Table 3:** Intra- and inter-day trueness (%relative error) and precision (%RSD) (n = 5).

Level	Nominal Concentrations (µM)	Intra-Day		Inter-Day	
		Trueness	Precision	Trueness	Precision
		(%RE)	(%RSD)	(%RE)	(%RSD)
LLOQ	0.25	−1.10%	10.90%	3.10%	9.60%
Low	1.0	4.80%	3.10%	0.80%	8.30%
Medium	5.0	1.30%	2.00%	1.40%	4.00%
High	25.0	1.10%	2.40%	1.30%	3.00%

**Table 4:** TMAO and lipidemic profile of healthy and hyperlipidemic volunteers.

Age	Sex	BMI	TMAO (µM)	TC (mg/dL)	TG (mg/dL)	HDL (mg/dL)	LDL (mg/dL)	CRP (mg/L)
(A) Healthy volunteers (University of the Aegean)								
21	F	21.0	0.666	149	55	69	81	0.04
21	F	19.8	0.725	151	64	72	79	0.07
20	M	23.2	0.779	122	84	46	69	0.18
20	F	18.6	0.827	144	76	54	85	0.07
29	F	27.2	0.879	159	83	61	91	0.11
23	F	18.7	1.005	141	97	61	71	0.03
23	F	20.8	1.425	173	45	96	65	0.09
30	F	26.0	1.736	166	75	70	96	0.07
23	M	22.4	2.131	179	124	53	101	0.38
22	F	24.0	2.463	156	68	61	76	0.25
22	F	23.1	2.705	171	69	70	82	0.24
24	M	25.6	2.882	154	58	69	73	0.17
(B) Hyperlipidemic volunteers (University Hospital of Ioannina)								
71	M	27.4	4.915	142	142	32	71	0.40
55	M	28.6	5.342	201	111	53	126	0.20
22	F	23.1	4.046	244	88	72	154	0.10
57	F	27.3	3.553	164	147	50	85	0.20
34	M	22.1	3.950	288	204	50	198	0.40
71	F	29.8	11.847	185	186	48	100	0.40
35	M	34.9	10.584	285	77	53	216	0.30

**Table 5:** Statistical analysis and comparison of healthy and hyperlipidemic volunteer groups.

Group	Value	Age	BMI	TMAO (µM)	TC
					(mg/dL)
Healthy	Mean	34	22.5	1.519	155
	SD	3	2.7	0.798	15
Hyperlipidemic	Mean	49	27.6	6.177	216
	SD	18	4.0	3.3306	54
	*p*	0.0015	0.0130	0.0002	0.0171
**Group**	**Value**	**TG** **(mg/dL)**	**HDL** **(mg/dL)**	**LDL** **(mg/dL)**	**CRP** **(mg/L)**
Healthy	Mean	75	65	81	0.140
	SD	20	12	11	0.100
Hyperlipidemic	Mean	136	51	136	0.290
	SD	44	11	52	0.110
	*p*	0.0018	0.0287	0.0221	0.0118

## Data Availability

The data presented in this study are available on request from the corresponding author.

## Supplementary Materials

The following supporting information can be downloaded at: https://www.mdpi.com/article/10.3390/molecules30112398/s1, Figure S1. Calibration curve of TMAO (0–25 μΜ) shows a linearity with a goodness of fit of R2 = 0.9995 (Bruker EVOQ ER System). Figure S2. A: Total Ion Count (TIC) Chromatograms of blank sample [1], blank sample with IS [2], and 0.25 μM sample with IS [3], B: all three TIC chromatograms overlaid. Figure S3. Determining TMAO on a Waters Xevo G2-XS QToF system. Figure S4. Calibration curve of TMAO (0–25 μΜ) on a Waters Xevo G2-XS QToF system.
