# Supplementary material for: Development and Validation of a Simple and Cost-Effective LC-MS/MS Method for the Quantitation of the Gut-Derived Metabolite Trimethylamine N-Oxide in Human Plasma of Healthy and Hyperlipidemic Volunteers
Source: Molecules. 2025 May 30;30(11):2398. doi: 10.3390/molecules30112398 (PMC12156282; doi:10.3390/molecules30112398)
Supplement: Supplementary file 1 [file molecules-30-02398-s001.zip › molecules-3592688-supplementary.pdf]

SUPPLEMENTARY MATERIAL

Development and validation of a simple and cost-effective LC-MS/MS method for the quantitation of the gut-derived metabolite Trimethylamine N-oxide in human plasma of healthy and hyperlipidemic volunteers.

Nikolaos A. Parisis[1], Panoraia Bousdouni[2], Aikaterini Kandyliari[2,3], Maria-Helen Spyridaki[4], Amalia Despoina Koutsogianni[5], Christina Telli[5], Konstantinos K. Tsilidis[6], Antonios E. Koutelidakis[2], Andreas G. Tzakos[1,7*]

1 Department of Chemistry, University of Ioannina, Ioannina, 45110, Greece
2 Department of Food Science and Nutrition, University of the Aegean, Lemnos, Myrina, 81400, Greece
3 Department of Food Science and Human Nutrition, Agricultural University of Athens, Athens, 11855, Greece
4 General Chemical State Laboratory, Chemical Service of Peloponnese, Western Hellas and Ionian Islands, Department of Chemical Services of Corfu, Greece
5 Department of Internal Medicine, Faculty of Medicine, School of Health Sciences, University of Ioannina, Greece.
6 Department of Hygiene and Epidemiology, University of Ioannina School of Medicine, Ioannina, Greece
7 Institute of Materials Science and Computing, University Research Center of Ioannina (URCI), Ioannina, Greece

* Correspondence: atzakos@uoi.gr; Tel.: +30-2651-008387

Supplemental Figures

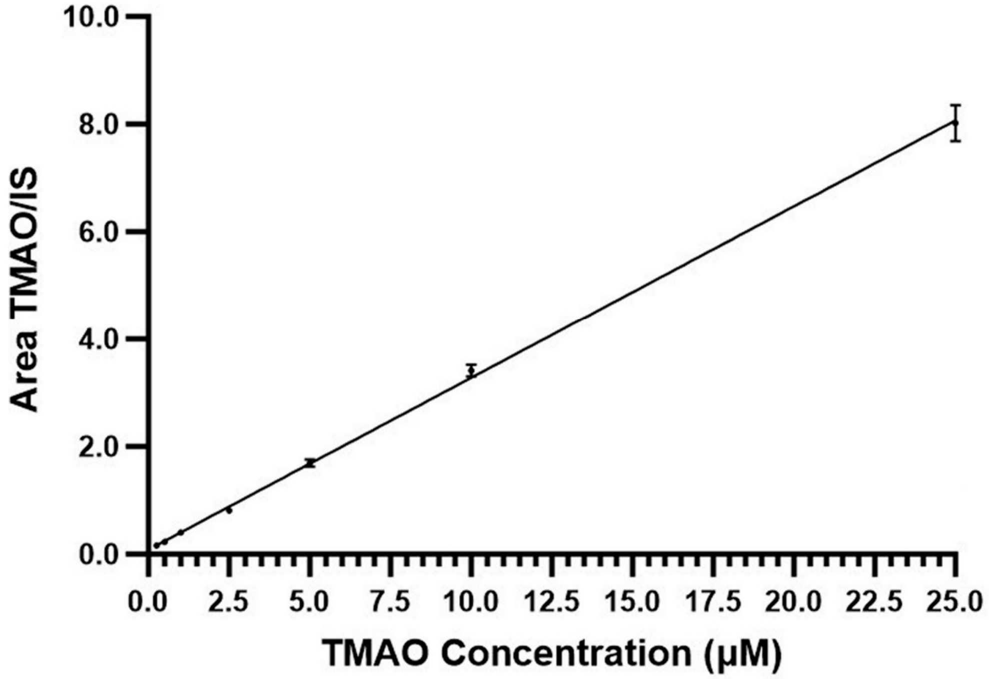

Figure S1. Calibration curve of TMAO (0-25µM) shows a linearity with a goodness of fit of R2 = 0.9995 (Bruker EVOQ ER System)

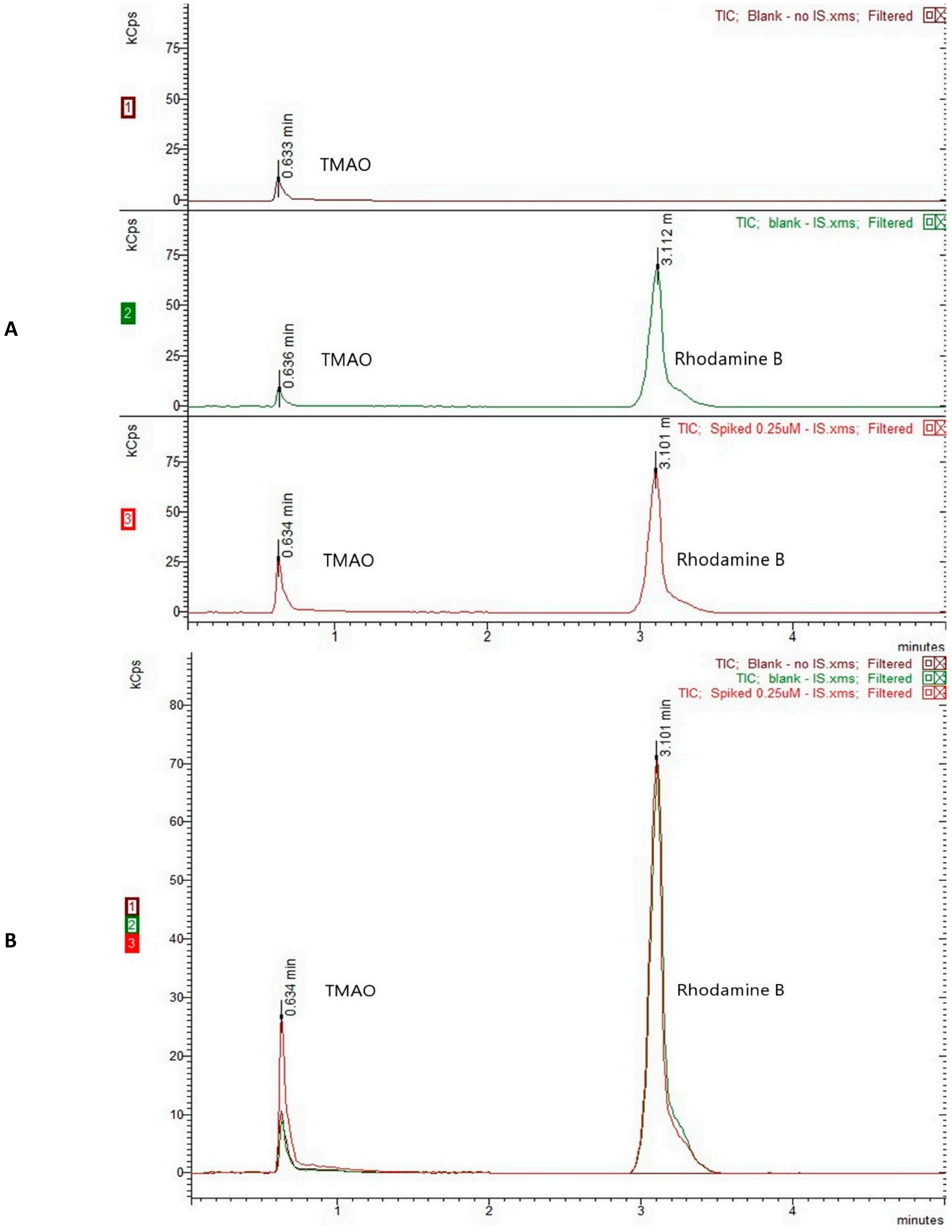

Figure S2. A: Total Ion Count (TIC) Chromatograms of blank sample [1], blank sample with IS [2], and 0.25 μM sample with IS [3], B: all three TIC chromatograms overlaid.

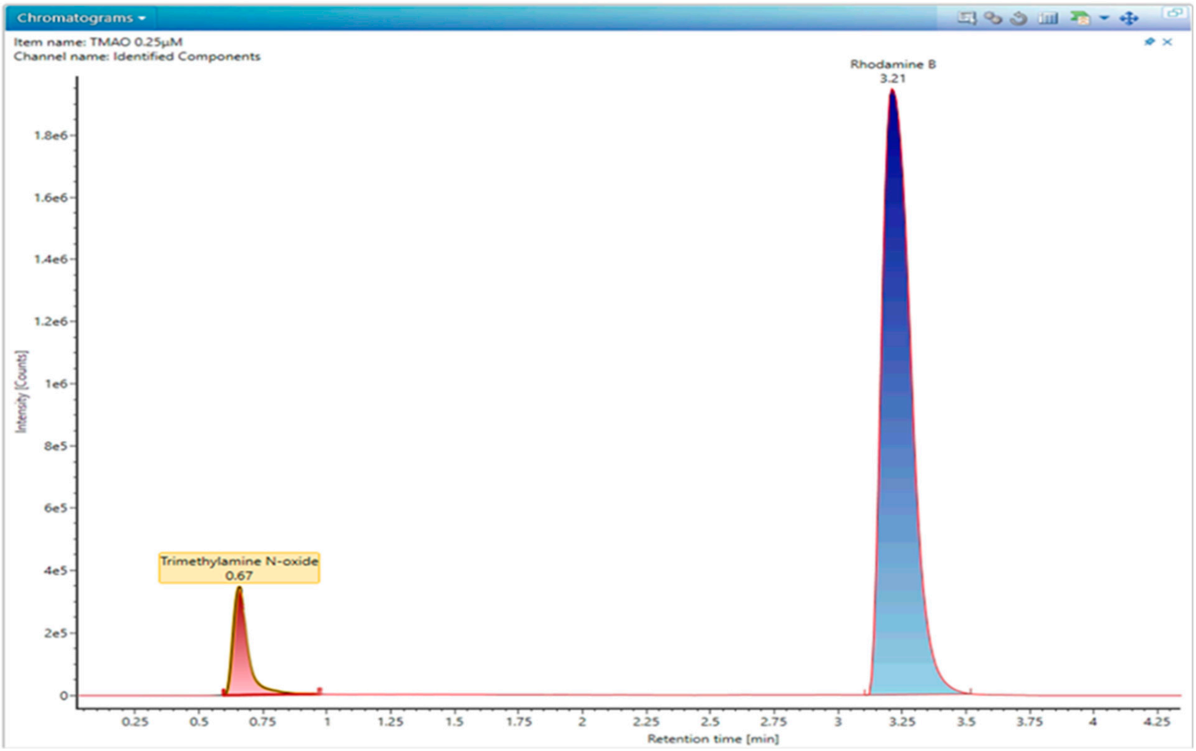

Figure S3. Determining TMAO on a Waters Xevo G2-XS QToF system.

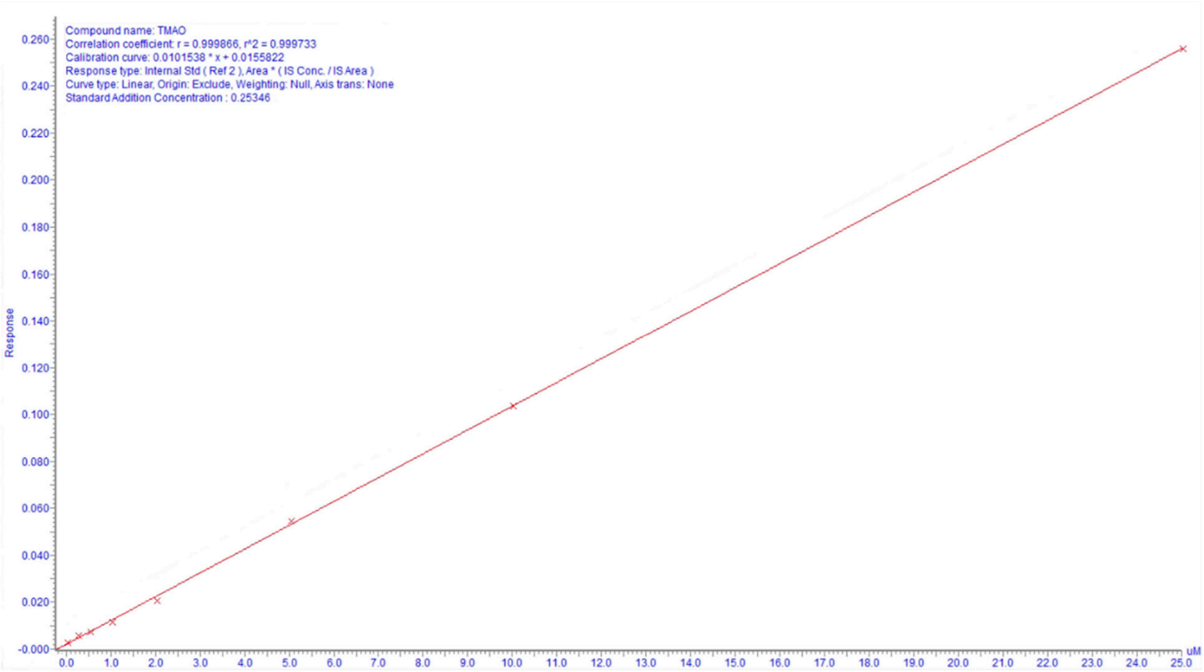

Figure S4. Calibration curve of TMAO (0-25µM) on a Waters Xevo G2-XS QToF system.
